# Okra (*Abelmoschus Esculentus*) as a Potential Dietary Medicine with Nutraceutical Importance for Sustainable Health Applications

**DOI:** 10.3390/molecules26030696

**Published:** 2021-01-28

**Authors:** Abd Elmoneim O. Elkhalifa, Eyad Alshammari, Mohd Adnan, Jerold C. Alcantara, Amir Mahgoub Awadelkareem, Nagat Elzein Eltoum, Khalid Mehmood, Bibhu Prasad Panda, Syed Amir Ashraf

**Affiliations:** 1 Department of Clinical Nutrition, College of Applied Medical Sciences, University of Hail, Hail 2440, Saudi Arabia; ao.abdalla@uoh.edu.sa (A.E.O.E.); eyadhealth@hotmail.com (E.A.); mahgoubamir22@gmail.com (A.M.A.); nagacademic0509@gmail.com (N.E.E.); 2 Department of Biology, College of Science, University of Hail, Hail 2440, Saudi Arabia; drmohdadnan@gmail.com; 3 Department of Clinical Laboratory Sciences, College of Applied Medical Sciences, University of Hail, Hail 2440, Saudi Arabia; jerold.alcantara@yahoo.com; 4 Department of Pharmaceutics, College of Pharmacy, University of Hail, Hail 2440, Saudi Arabia; adckhalid@gmail.com; 5 Microbial and Pharmaceutical Biotechnology Laboratory, Centre for Advanced Research and Pharmaceutical Sciences, School of Pharmaceutical Education and Research, Jamia Hamdard, New Delhi 110062, India; bppanda@jamiahamdard.ac.in

**Keywords:** antidiabetic, cardioprotective, functional foods, nutraceuticals, okra, phytotherapy

## Abstract

Recently, there has been a paradigm shift from conventional therapies to relatively safer phytotherapies. This divergence is crucial for the management of various chronic diseases. Okra (*Abelmoschus esculentus* L.) is a popular vegetable crop with good nutritional significance, along with certain therapeutic values, which makes it a potential candidate in the use of a variety of nutraceuticals. Different parts of the okra fruit (mucilage, seed, and pods) contain certain important bioactive components, which confer its medicinal properties. The phytochemicals of okra have been studied for their potential therapeutic activities on various chronic diseases, such as type-2 diabetes, cardiovascular, and digestive diseases, as well as the antifatigue effect, liver detoxification, antibacterial, and chemo-preventive activities. Moreover, okra mucilage has been widely used in medicinal applications such as a plasma replacement or blood volume expanders. Overall, okra is considered to be an easily available, low-cost vegetable crop with various nutritional values and potential health benefits. Despite several reports about its therapeutic benefits and potential nutraceutical significance, there is a dearth of research on the pharmacokinetics and bioavailability of okra, which has hampered its widespread use in the nutraceutical industry. This review summarizes the available literature on the bioactive composition of okra and its potential nutraceutical significance. It will also provide a platform for further research on the pharmacokinetics and bioavailability of okra for its possible commercial production as a therapeutic agent against various chronic diseases.

## 1. Introduction

Okra (*Abelmoschus esculentus* L.), belonging to the family *Malvaceae*, is commonly known as Lady’s finger, as well as by several vernacular names, including okra, bhindi, okura, quimgombo, bamia, gombo, and lai long ma, in the different geographical regions of its cultivation [1]. Okra is believed to have originated near Ethiopia, where it was frequently cultivated by the Egyptians during the 12th century, and thereafter spread throughout the Middle East and North Africa [2,3]. Okra is an annual shrub that is cultivated mostly within tropical and subtropical regions across the globe and represents a popular garden crop, as well as a farm crop. It is a widely cultivated food crop and is globally known for its palatability. The immature green pods of okra are usually consumed as vegetables, while the extract of the pods also serves as a thickening agent in numerous recipes for soups, as well as sauces, to augment their viscosity [4,5]. Another noteworthy application of okra fruit is their wide use in the pickle industry. The polysaccharides present in okra are used in sweetened frozen foods such as icecreams, as well as bakery products, due to their health benefits and longer shelf-lives [6,7,8]. Anatomically, the fruits, stem, and leaves of okra are covered with minute soft, hairy structures. Although the flowering of the okra plant is perennial, it is highly dependent on various biotic and abiotic factors. The leaves of okra are polymorphous, characterized by hairy upper and lower surfaces, whereas the petioles are around 15 cmlong. The flowers of okra can be easily recognized due to their slight yellowish color with a crimson center. The edible part of okra or its capsule (pod) measures approximately 15–20 cm in length and has a pyramidal-oblong, pentagonal, hispid appearance. Historically, okra pods were utilized for various purposes, such as in food, appetite boosters, astringents, and as an aphrodisiac. Furthermore, okra pods have also been recommended to cure dysentery, gonorrhea, and urinary complications [9]. Extracts of young okra pods have also been reported to display moisturizing and diuretic properties, whereas the seeds of this plant have been reported to possess anticancer and fungicidal properties [10].

Recently, okra has been used not only for its nutritional values but, also, for its nutraceutical and therapeutic properties, owing to the presence of various important bioactive compounds and their associated bioactivities. This review presents a summary of the nutritional significance of okra, as well as the possible pharmacological applications of okra bioactive components, and to explore the possible characteristics forthe development and formulation of nutraceuticals andfunctional food. In addition, this review also focuses on the nutraceutical potential of *Abelmoschus esculentus* for various therapeutic purposes, as well as to demonstrate the benefit of okra-based nutraceuticals and their consumption.

## 2. Nutritional and Bioactive Constituents in Okra

Okra is probably a proficient dietary constituent rather than a staple food crop. Small industries (Surajbala Exports Private Limited, New Delhi, India and Hunan QiyiXinye culture media, Hunan, China) have utilized okra seeds for oil extraction [11,12]. Additionally, the lipid content of any food item is considered as one of the important aspects of itsnutritional value, andseveral food types have different levels oflipid contents, including triacylglycerols, polar lipids, free fatty acids, or diacylglycerols. Among these constituents, fatty acids are largely responsible for determining the stability and nutritional value of food types. Triacylglycerols are biomolecules that are composed of unsaturated and saturated fatty acids with subtle differences in the number of associated acyl group repeats, along with the repetitions and positions of double bonds. Importantly, these lipids are naturally destined to be energy reservoirs [13].

Earlier, Savello et al. reported that the seeds of the okra plant represent a rich source of oil, constituting 20 to 40% of the total composition, which varies with the extraction procedure [14]. Linoleic acid, a well-known representative of polyunsaturated fatty acids (PUFA), is the dominant constituent of the oil content (47.4%) of okra seeds [14]. Other important dietary constituents essential for human growth are the amino acids and their polymers, viz., proteins [15,16]. Okra seeds have been reported to have different protein compositions from cereals and pulses, as their protein ingredients are modified to bear a balance of characteristic amino acids, namely lysine and tryptophan. Thus, owing to their rich content of essential amino acids, okra seeds represent an important constituent of the human diet [17]. Okra also serves as a potentially rich source of vitamins and carbohydrates, which are also regarded as a vital nutritional components of the diet [18]. Okra pods are also reported for their rich nutritional compositions and are frequently consumed either after boiling, frying, or cooking [19]. The nutritional values of different edible parts of okra (per a 100 g serving) are mentioned in Table 1.

Okra also provides a rich supply of minerals required for maintaining normal homeostasis at the cellular level. The edible plant parts have been documented to possess calcium (Ca), phosphorus (P), and iron (Fe) at different amounts of 84, 90, and 1.20 mg, respectively. It also possesses a β-carotene, riboflavin, and vitamin B complex at the approximate concentrations of 185 µg, 0.08 mg, and 0.04 mg, respectively [21]. The other compositional vitamins of okra plants are mentioned in Table 2.

The mucilage from the okra plant is primarily constituted by carbohydrates [22]. Furthermore, the young pods of okra plants are composed of polysaccharides (Mw~170,000), along with 11% amino acids. Moreover, okra pods are primarily constituted by equivalent amounts of galactose and galacturonic acid (25 and 27%, respectively), along with 22% rhamnose. In some parts of the world, especially West Africa, okra pods are also consumed in a dried form after the addition of other ingredients. However, one major nutritional drawback of such consumption is the absence of β-carotene or retinol (vitamin A) in a dried form [23]. Moreover, fresh pods of okra also provide viscous dietary fiber, which has been reported to minimize cholesterol levels. In previous studies, the maximum concentrations of nutrients were reported from okra pods that were aged only up to seven days [24,25].

## 3. The Pharmacological and Potential Applications of Okra-Derived Biomolecules

### 3.1. Antidiabetic Efficacy

The incidences of metabolic disorders, particularly diabetes, have tremendously increased in populations throughout the world. Although diabetes is caused by several factors, oxidative imbalance and inflammatory responses have been identified as their most common repercussions [26,27]. Recently, type 2 diabetes patients have also been reported to be associated with obesity and high lipid profiles [28]. Okra plant parts have been widely reported to reduce hyperglycemic levels. Okra mucilage, along with ethanolic and aqueous extracts of the pods, have been reported to lower the glucose levels in the blood of alloxan-induced diabetes models [29,30]. Diabetes nephropathy (DN) is a common complication of diabetes that has become a serious threat to human health and is expected to become the common cause of end-stage renal disease and cardiovascular events [31,32,33].

In clinical practice, a Chinese single plant-based drug extracted from the dry flowers of *Abelmoschus manihot*, named the Huangkui capsule (HKC), is used for the treatment of CKD (chronic kidney disease), DN, chronic glomerulonephritis, membranous nephropathy, and other inflammatory diseases. It is a patented drug approved by the State Food and Drug Administration of China (Z19990040) in 1999 for diabetes-related complications [34,35,36].

In a rat model of unilateral nephrectomy and doxorubicin-induced nephropathy, a HKC dose of 0.5 and 2 g/kg is administered via an intragastric (IG) manner for 28 days. It was found that the general status of the rat improved, as alleviated renal histological changes, proteinuria, albuminuria, glomerulosclerosis, and a decreased infiltration of ED1+ and ED3+ macrophages into the glomeruli were noticed. An inhibition of the protein expression of tumor necrosis factor (TNF)-α in the kidney was found. Further studying of the mechanism shows that, in a rat model of doxorubicin-induced nephropathy, HKC can downregulate the protein expression of transforming growth factor (TGF)-β1 and the p38mitogen-activated protein kinase (MAPK) by suppressing the p38/ MAPK signaling pathway [37,38].

Additionally, within a rat model of DN induced by unilateral nephrectomy and streptozotocin (STZ) injections rather than lipoic acid, it was noted that, when HKC was administered (IG) at doses of 0.75 and 2.0 g/kg for 56 days, the urinary albumin levels were reduced. It also improves renal function, as it decreases the blood urea nitrogen (BUN) and serum uric acid levels. The number of cells and the extracellular matrix of glomeruli is reduced to alleviate kidney fibrosis by HKC and reverse the increase in the markers of oxidative stress, such as malondialdehyde (MDA), 8-hydroxy-2′-deoxyguanosine, total superoxide dismutase (SOD), and nicotinamide adenine dinucleotide phosphate oxidase-4 [39]. Further studies of the mechanism proved that HKC simultaneously decreased the protein expression of pp38MAPK, p-Akt, TGF-β1, and TNF-αby inhibiting the p38MAPK and Akt signaling pathways in the kidney in a rat model of DN.

Later, in vitro and in vivo studies indicated that an increase in the mRNA expression of peroxisome proliferator-activated receptor (PPAR)-α and PPARG in the livers and kidneys of rats with DN was observed when HKC was administered (IG) at doses of 75, 135, and 300 mg/kg for 84 days. It was also found that HKC administration also increased the serum albumin levels, while the serum triglycerides, cholesterol, and total fats levels were lowered in a dose-dependent manner. This result was seen in the livers of rats with DN as compared to irbesartan [33]. Moreover, HKC decreased the expression of interleukin (IL)-1, IL-2, IL-6, and TNF-α by suppressing the inflammatory reaction in the kidneys of rats with DN. Strikingly, HKC alleviated endoplasmic reticulum stress and decreased the activation of the c-Jun NH2-terminal kinase in the livers and kidneys of rats with DN and, subsequently, reduced renal injury [33].

The results obtained from the above studies demonstrated that HKC can be a potential agent for DN treatment in humans. It was found that, when HKC was administered via IG at a dose of 0.75 g/Kg for 28 days, a significant decrease in the levels of BUN, serum creatinine, and urine protein in the plasma was found. The molecular mechanisms demonstrated that HKC notably downregulated the protein expression of NADPH oxidase (NOX)-1, NOX-2, NOX-4, α-smooth muscle actin (αSMA), and the p-extracellular signal-regulated kinase (ERK)1/2 by inhibiting the NADPH oxidase/reactive oxygen species (ROS)/ERK signaling pathways in renal tissue in rats with chronic renal failure induced by adenine in vivo [40]. Subsequent phytochemical investigations have shownthat the main bioactive components of HKC are quercetin, quercetin-3′-O-glucoside, isoquercitrin, and hyperoside. A 100-µM concentration of gossypetin-8-*O*-β-D-glucuronide can inhibit the protein expression of smooth muscle actin, p-ERK1/2, NOX-1, NOX-2, and NOX-4 in HK-2 cells, which are induced by high glucose levels in the same way as the NOX inhibitor diphenyleneiodonium [40].

Furthermore, aqueous extracts from the pods of okra plants co-administered with metformin also resulted in hypoglycemia in Long Evans rats [41]. The enzyme α-amylase, which acts by breaking polysaccharides, resulting in the availability of glucose, has been considered as a vital enzyme for fulfilling the energy requirements of the human body. Water-soluble seed and peel extracts from okra plants have also been previously reported to inhibit the activities of both α-glucosidase and α-amylase [42]. Moreover, Lu and coworkers reported that the α-glucosidase and α-amylase inhibitory activities of premature seeds of the okra plant were due to oligomeric proanthocyanidins [43]. Further research on the antidiabetic efficacy of the okra plant suggested that rhamnogalacturonan also mediated the antidiabetic activity [44].

### 3.2. Antioxidant Efficacy

Okra pods are immature fruits of okra that are consumed in nearly all parts of the world as a vegetable. Previous studies have reported that immature okra pods have antioxidant potential [45,46]. In elaboration of this, a recent study elucidated that the antioxidant efficacy of okra pods may be due to the large amounts of polyphenols (29.5%) present within the seeds of immature pods. After the ingestion of food or a beverage, flavonoids in the ingested matrix must pass from the gut lumen into the circulatory system in order to be absorbed. Since, in plants, almost all flavonoids are in the form of glycosides, the attached sugar must be removed following consumption before absorption can take place [47]. These polyphenols from immature okra pods carry out the antioxidant activity by lowering the MDA level and increasing the SOD and glutathione peroxidase (GSH-Px) levels [48,49]. Flavonoids are a large group of secondary plant metabolites and occur as either aglycones or conjugates with glycosides and acyl groups, wherein around 8000 different types have been identified so far [50]. The major phenolic compounds found in okra fruits are quercetin-3-O-gentiobioside, quercetin-3-O-glucoside (isoquercitrin), rutin, a quercetin derivative, protocatechuic acid, and a catechin derivative, of which quercetin-3-O-gentiobioside was the most abundant phenolic compound [51,52]. The major contributor of the antioxidant capacity is quercetin-3-O-gentiobioside. It also exhibits inhibitory effects on digestive enzymes like lipase, α-glucosidase, and α-amylase [48,51].

The free radicalscavenging and ferric-reducing capabilities of okra pods have been documented in previous studies. In one study, okra extract obtained by cold extraction and boiling the fruit in water showed notable antioxidant activity [53]. Seeds from the okra plant are also a rich source of phenols, namely procyanidin B1 and B2, both of which are involved in DPPH (1,1-diphenyl-2-picrylhydrazyl) and ABTS (2,2′-azino-bis(3-ethylbenzothiazoline-6-sulfonic acid) free radicalscavenging activities [54]. Liao et al. also reported the antioxidant and ferric-reducing activities of okra pods, although they identified two specific glucopyranoside compounds, 5,7,3′,4′-tetrahydroxy-4″-O-methylflavonol-3-O-β-d-glucopyranoside and 5,7,3′,4′-tetrahydroxy flavonol−3-O-[β-d-glucopyranosyl-(1→6)]-β-d-glucopyranoside, to be the responsible bioactive compounds [55]. Subsequently, it was also reported that alternative parts of okra—namely, the flowers, leaf, seed, and pods—also had substantial antioxidant efficacy [55]. In a desirability study, a powder of the seed and peel of okra augmented the levels of hepatic, renal, and pancreatic SOD and glutathione peroxidase in streptozotocin models of diabetes. A similar treatment regime also resulted in reduced levels of glutathione and thiobarbituric acid [41]. Similar observations were also recorded by Doreddula et al., in which extracts from okra seeds at concentrations in the range of 100–250 µg/mL showed substantial antioxidant effects through ferric reduction, β-carotene-linoleic assay, and DPPH. Moreover, different fractions of okra plants have also been reported to reduce malondialdehyde and elevate the levels of glutathione peroxidase and superoxide dismutase [51].

### 3.3. Anticancer Effect

Cancer is the second-leading cause of death globally, and despite the advances in drug development, it is still necessary to develop new plant-derived medicines. There is an urgent need for new anticancer drugs, because cancerous cells are developing resistances against the currently available drugs, like vinca alkaloids and taxanes [56,57].

The term cancer represents a broad group of malignancies showing the key characteristic of uncontrolled proliferation, aided by various regulatory and functional changes, which, in turn, ensure a systemic spread throughout the body [58]. Although scientific cancer research communities have made remarkable progress in understanding the mechanisms responsible for such a debilitating disease, an effective treatment strategy without accompanying toxic effects has yet to be made available. The therapeutic management of such life-threatening modalities should focus on the increased exploration of a chemo-preventive approach through plant-based natural agents from different sources. To this end, plant products have attracted several researchers across the world because of their selective toxicity against malignant cells [59,60,61]. It is also reported that the flowers of okra contain substantial amounts of flavonoids and phenols, as compared to the pods, peel, leaves, and seeds [62,63]. A recent report elucidated that purified fractions of flavonoids from the flowers of okra plants had a significant antitumor effect on colorectal malignancy both in-vitro, as well as in vivo, exerting a strong antioxidant potency concomitantly with substantial antiproliferative effects on tumor growth. The antiproliferative effect of flavonoids within okra flowers induced the activation of p53, culminating into the ceasing of mitochondrial functions within colorectal tumor cells, ultimately resulting in apoptosis and restraining the autophagy [64].

Subsequently, the anticancerous effect of okra seed extracts have also been documentedin vitrothrough several other cell lines. The flavonoid constituents of seed extracts showed enhanced cytotoxic effects on human-derived breast cancer cells (MCF-7) in comparison with hepatoma cells of human origin (HepG2) and human cervical cancer (HeLa) cells in a dose-dependent manner. These observations affirmed that the flavonoid isoquercitrin, in a synergistic association with other flavonoids, inhibited vascular endothelial growth factor (VEGF), resulting in the apoptosis of cancerous cells [65]. Additionally, Hyperin—also known as quercetin-3-O-β-d-galactoside—is an important flavonoid constituent of okra. Hyperin has also been reported for its anticancerous potential in gastric cancer cells (CHI) by establishing an antiproliferative, antimigratory, and anti-invasive environment resulting in apoptosis by blocking the Wnt/β-catenin signal pathway [66].

Carbohydrate-binding proteins such as lectins have been widely investigated for their anticancer effects [67,68]. Lectins from okra have been documented to activate caspase-mediated downstream signaling in MCF7 cells and normal fibroblasts (CCD-1059 SK) [69]. Furthermore, the extract of okra pulp has also been attached to gold nanoparticles and observed to exhibit a significant induction of oxidative stress, followed by the depolarization of mitochondrial membrane potential and apoptosis in Jurkat cells, thereby indicating its anticancer potential [70]. However, studies focusing on the capability of okra and its parts to reduce cancer progression and its associated effects are rare.

### 3.4. Immunomodulatory Potential

The immune system acts as a well-defined protective shield against noxious external or internal intervening agents. The immune system plays a critical role in protecting the human body from infectious diseases. Its two main contributors include innate and acquired immunity responses. The most important feature of innate immunity is its lack of specific recognition. This type of immune system responds to all pathogens, regardless of their nature. Innate immunity is composed of immune and nonimmune components, whilst acquired immunity has only immune elements. Phytochemicals are the naturally occurring secondary metabolites present in abundance in fruits and vegetables. They do not have any nutritional importance, but they are essential for the growth and maintenance of plants, and with evolution, humans have learnt the ways to harvest and manipulate these phytochemicals for their own benefits [71,72].

The biologically potent constituents of *A. esculentus* have also been reported to modulate the complex immune system. The administration of lectin from okra in mice at low concentrations (0.01, 0.1, and 1 mg/kg) has demonstrated significant inflammatory effects [45]. Recently, the administration of the ethanolic extract of okra lowered inflammation in Wistar rats subjected to acute gastric mucosal injury [73]. The polysaccharide constituents of an aqueous okra extract were demonstrated to augment the hemoglobin content and expression of major histocompatibility complex (MHC) II and CD80/89 within the bone marrow hematopoietic cells derived from rats, as well as reduce endocytosis. Furthermore, the aqueous extracts also upregulate the expression of interleukin-12 and interferon-γ, along with the simultaneous reduction of the anti-inflammatory cytokine interleukin-10 [74]. Macrophages are key components of innate immunity and act as a prerequisite for the effective functioning of the innate immune systems. The immunomodulatory effects of polysaccharides from okra have also been evaluated on macrophage cell lines by Chen et al. (2016), who reported an increase in nitric oxide (NO) synthesis, inducible NO synthase (iNOS) expression, and the levels of tumor necrosis factor-α and cytokines in RAW264.7 cells following treatment [75].

### 3.5. Microbicidal Action

**Figure 1:**
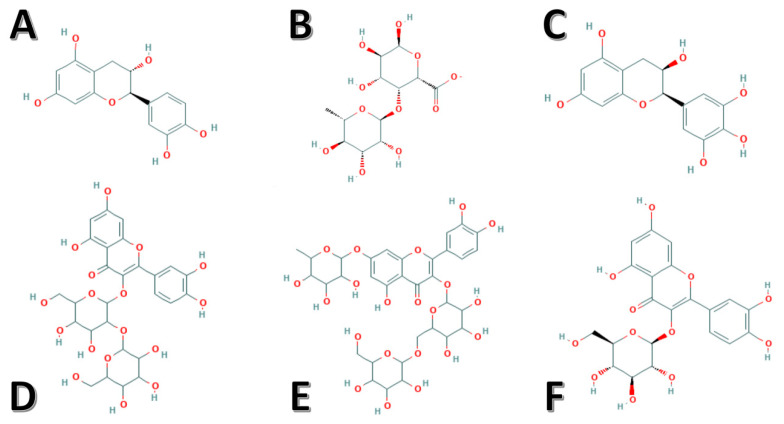
Chemical structures of the identified potent bioactive components derived from okra. (**A**) Catechin, (**B**) rhamnogalacturonan, (**C**) epigallocatechin, (**D**) quercetin-3-O-sophoroside, (**E**) Quercetin-3-O-[glucosyl(1->6) glucoside]-7-O-rhamnoside, and (**F**) isoquercitrin.

The lipid content—namely, palmitic and stearic acids of lyophilized extracts from okra pods—along with its aqueous counterpart, have earlier been reported to inhibit *Rhodococcus opacus*, *Rhodococcus erythropolis*, *Mycobacterium aurum*, *Escherichia coli*, *Staphylococcus aureus*, *Pseudomonas aeruginosa*, and *Xanthobacter* Py2 [76]. Moreover, the gold nanoparticles synthesized from the okra extract (pulp) also displayed substantial microbicidal efficacy against *Bacillus cereus*, *Bacillus subtilis*, *E. coli*, *P. aeruginosa*, and *M. luteus* [77]. Furthermore, fractionsof the pods from okra that are rich in carbohydrates were also documented for their activity against *Helicobacter pylori* [78]. Apart from the above-mentioned therapeutic effects of okra fruits, some therapeutic effects and their mechanisms of action are presented below in Table 3, alongwith the presentation of various bioactive components present in okra and their chemical structures (Figure 1).

## 4. In Vivo Studies on the Health Benefits of Okra and Its Components

As stated earlier in this review, okra and its bioactive components are reported to have potential beneficial health effects. A significant reduction in the blood glucose level, along with an increase in body weight, was reported by Dubey and Mishra (2017) and Sabitha et al. (2011) in streptozotocin-induced diabetic rats when fed the peel and seed powder of okra.Sabitha et al. also reported a significant increased level of hemoglobin and the total protein level and a decrease in HbA1c. They also reported that okra peel and seed powder at a dose of 200 mg/kg showed a significant reduction inblood glucose compared to a 100 mg/kg dose. Additionally, the treatment of both the doses of okra seed powders significantly produced a greater blood glucose reduction when compared to okra peel powderat a 100 or 200-mg/kg dose. Meanwhile, there several studies suggested multiple mechanisms of antidiabetic plants to exert their blood glucose-lowering effects, such as the inhibition of carbohydrate metabolizing enzymes, enhancement of insulin sensitivity, regeneration of damaged pancreatic islet β-cells, and enhancement of insulin secretion and release [41,94]. Okra polysaccharides were also reported to lower the body weight and glucose levels, improve the glucose tolerance, and decrease the total serum cholesterol levels in mice fed with a high-fat diet. Different studies reported the effects of different parts of okra on alloxan-induced diabetic rats and showed that there was a significant reduction in the blood glucose level and glycated hemoglobin and an improvement in the lipid profile compared with the diabetic nontreated control rats and comparable with the metformin-positive control group [95,96]. Different parts of okra fruits can stimulate glycogen synthesis in the liver and delay the intestinal absorption of glucose in alloxan-induced diabetic rats [97]. In the same study, the histopathological examination of the pancreatic tissue after the administration of okra fruits revealed the evidence of pancreatic islet cell regeneration [97]. Okra supplementation statistically reduced the highlevels of fasting blood sugar, total cholesterol, and triglycerides and decreased the homeostasis model assessment of the basal insulin resistance index in diabetic rats, as reported by Majd et al. (2018) [98].

Nguekouo et al. (2018), in an in vivo study, showed that boiling and roasting do not change the antidiabetic potential of okra fruits and seeds [99]. Ortaç et al. (2018) reported in an in vivo study that okra has a gastroprotective effect against ethanol and could decrease a gastric ulcer, as indicated by the biochemical and histopathological data. They concluded that okra could be a possible therapeutic antiulcer agent [74]. Hossen et al. (2013) showed that the methanol extract of okra had a good central nervous system depressant activity, along with a high painkiller activity, on Swiss albino mice [100]. Wang et al. (2104) demonstrated that mice fed an okra diet execrated more cholesterol in their stools and had lower total blood cholesterol levels compared to the control mice group [101]. A four-year study conducted on 1100 people showed that people who ate a diet rich in polyphenols had lower inflammatory markers associated with heart disease, and as okra is one of the polyphenol-rich diets, okra may therefore help to protect from cardiovascular diseases [102]. Monte et al. (2014) reported that the lectin available in okra can stop cancer cell growth by up to 63% when they did a testtube study on breast cancer cells. Additionally, Vayssade et al. (2010), in a testtube study on metastatic mouse melanoma cells, showed that okra extract leads to cancer cell death [85]. Doreddula et al. (2014) revealed that the seed extracts of okra have an antioxidant, antistress effect in the bloodstream of mice [103].

## 5. Therapeutic Prospects of Okra as Dietary Medicine/Nutraceuticals

Around two-thirds of the world population (7.8 billion) is dependent on plant-based materials for their medicinal and healing properties, mainly because of their easy availability, accessibility, affordability, and safety, as well as the traditional beliefs of the consumers [104]. A very old quote by Hippocrates stated, “Let food be thy medicine and medicine be thy food”, which described the significance of food and its nutritional, as well as therapeutic, values for the prevention, treatment, and management of diseases [105]. Thereafter, DeFelice coined the term nutraceutical by merging “nutrition” and “pharmaceutical” and defined it as food or part of a food that not only imparts health benefits but, also, contributes to the prevention or treatment of various diseases [106]. Importantly, nutraceuticals have been formulated in such a way that they could benefit or facilitate the management of human health without instigating any harm due to their natural occurrence. Nutraceuticals derived from plants, animals, or live microorganisms possess great potential for use by scientific communities, food researchers, and food-processing industries to produce unique foods or food components for the forthcoming needs of human beings to stay healthy without any side effects. Currently, the rapid rise in demand for nutraceutical products has been largely observed because of their therapeutic value in various diseases, such as diabetes, hypertension, arthritis, inflammatory bowel disease, the common cold, dyslipidemia, heart disease, and cancer. Nutraceutical products may also increase the lifespan by delaying aging, promoting the integrity of the body, and sustaining smooth normal functioning [107]. Moreover, based upon various pharmacological potentials of okra-derived molecules, okra has been seen as one of the potential sources of nutraceuticals.

**Figure 2:**
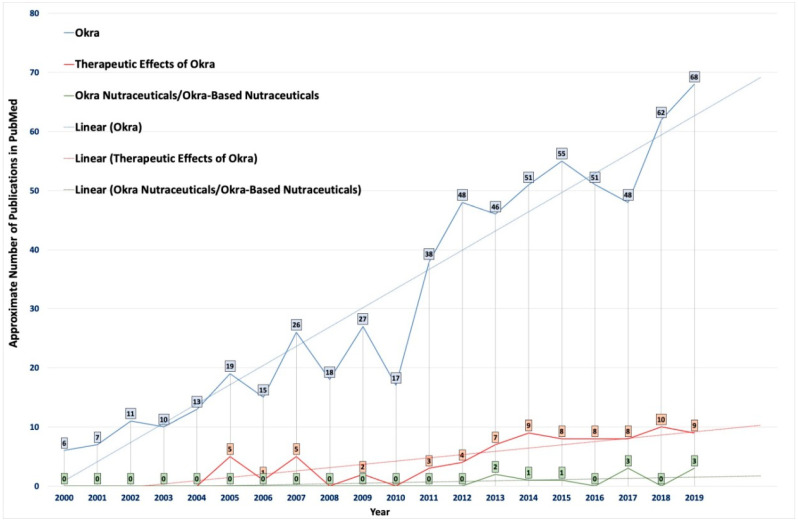
Statistical data showing the number of publications on the use of okra in pharmaceuticals indexed in PubMed from 2000 to 2019. The colors indicate publications available in PubMed after using the following keywords/phrases: (1) okra (blue), (2) therapeutic effect of okra (orange), and (3) okra nutraceuticals/okra-based nutraceuticals (green).The average trendlines show the importance and urgent need for research concerning the development of easily available okra (or its by-products)-based nutraceuticals and functional foods.

It was observed that there was an increase in the number of studies investigating the therapeutic value of okra (Figure 2). The phytochemicals present in okra have been suggested to have potential applications for the treatment and management for various diseases (Table 3 and Table 4) [9]. However, the potential of this extraordinary, cost-effective, cheap vegetable crop is still not fully used for its therapeutic or nutraceutical potential effects [9]. Therefore, there is an urgent need for such an easily available cheap vegetable crop like okra to be used in a nutraceutical formulation. Hence, okra-basednutraceuticals could play an essential role in the prevention and management of health, along with health improvements. Therefore, *A. esculentus,* an edible vegetable, could be an ideal source of nutraceuticals, since it contains both nutritionally active chemicals, as well as a source for various physiological advantages, as presented in Table 4. Few countries have traditionally used okra in folk medicine for various therapeutic purposes, such as gastroprotective, antiulcerogenic, and as a diuretic. The recent urbanization and changes in lifestyles, eating habits, and other factors have exposed the global population to a variety of chronic diseases. Since okra is an easily available and low-cost vegetable, it could potentially become an important nutraceutical product for populations in countries, irrespective of their stage of development. Particularly, okra-based nutraceuticals could become an ideal source of nutrition for people suffering from malnutrition in lesser-developed countries. However, the available studies in the literature have not specifically analyzed the therapeutic potential of okra as a nutraceutical.

## 6. Formulation and Development of Okra-Based Nutraceuticals

**Figure 3:**
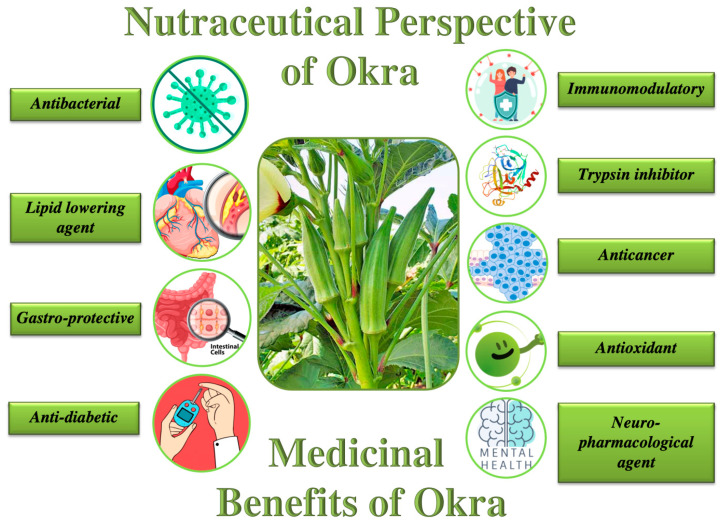
Illustrative representation of the okra-mediated beneficial effects that have been scientifically established to date.

Nutraceuticals are broadly described as food or parts of food that provide incremental health benefits. Okra-based nutraceuticals represent popular health foods, owing to its intrinsic nutritional and other bioactive components, which show health-associated beneficial properties (Figure 3) [133]. Several efforts are being made to improve the well-known hypoglycemic outcomes of okra fruit by formulating different proportions of seeds and peels of Ex-maradi Okra fruit in the ratio of (10:90, 20:80, 30:70, 40:60, 50:50%, and so on), which is subsequently followed by investigating the antidiabetic and antioxidant efficacy of these formulations in vitro. Recent findings have led to the conclusion that seeds and peels at the ratio of 10:90% are the most efficient in exhibiting substantial in vitro antidiabetic and antioxidant efficacy [134,135]. Subsequently, it was recommended that the nutraceutical formulation of peel and Ex-maradi okra seeds in the ratio (10:90) exhibits substantial hypoglycemic and hypolipidemic activity in alloxan models of diabetes (rodents) and was thus appropriate for further improvements for the formulation of okra-based nutraceutical interventions in diabetes mellitus [135]. On the other hand, okra polysaccharides have also been reported to inhibit human cancer cell proliferation [136]. This possibly indicates their potential usage as anticancer nutraceutical formulations. However, an individual’s susceptibility to any disease also largely depends upon genetic predisposition and lifestyle habits, such as smoking and high alcohol consumption. Therefore, the efficacy of nutraceuticals can vary from person to person. Nutraceuticals have proven health benefits, and their consumption (within their acceptable recommended dietary intakes) may help in the prevention of disease and allow humans to maintain overall good health. Therefore, since various parts of okra (fruit, seed, pulp, and mucilage) carry several therapeutic purposes, it can be considered to be an important vegetable crop for nutraceutical purposes.

## 7. Global Okra Production and Possible Nutraceutical Market

Okra, being an inexpensive popular vegetable crop, is consumed by several populations globally and is a local staple food in low-income countries. Nowadays, due to its nutritional and health benefits, there is a growing demand for okra, and different okra products are available for purchase on online marketplaces. Recently, the agency Market Research Future estimated that the global okra seed market could earn a revenue of USD 352.7 million and register a 9.8% compound annual growth rate during the period 2018–2023 [137]. Globally, the market of okra seeds is geographically largely divided into Europe, Asia-Pacific, and North America, followed by the remaining countries. In 2018, the largest accreditation for the contribution of the okra market share (63.77%) was recorded by the Asia-Pacific region. It is estimated that the okra-based nutraceutical market will reach a worth of 222.9 million USD by the end of the year 2023. Small-scale manufacturers are a major cause for the disintegration of the okra market in the Asia-Pacific region. Pakistan, Malaysia, India, and the Philippines are regarded as the dominant producers of okra seeds [19,137,138,139]. In recent times, India has been the prominent producer of okra globally, followed by the remaining countries mentioned above. Since 2018, these remaining countries have held a 33.0% share of the global okra market. This enhanced expansion within the local market is attributed to increased cultivation, as well as the development of genetically modified (GM) seeds. Furthermore, the acceptance of hybrid and disorder-resistant seeds within the region has also facilitated the noticeable expansion of the okra market. Africa is now predicted to globally dominate the market for the consumption of okra seeds. It represents approximately 69% of the territorial market share due to increased accessibility to more arable croplands within the country. On the other hand, during 2017, North America accounted for only 2.2% of the okra market share, whereas Europe accounted for only 1.0%. At the same time, Mexico is known to be a dominant producer of okra in North America because of the high cultivation of okra within the country [109]. The global okra seed-mediated market (OSM) is divided categorically and regionally. Based on the category, the OSM is further divided into conventional and organic seeds of okra plants. The conventional OSM (cOSM) is more prominent, with a market share of 90.5% since 2018. The market dominance of cOSM could be attributed to the exploitation of different varieties, namely open-pollinated and traditional. In contrast, organic OSM is estimated to show a high growth rate of 10.7%, which could be attributed to a shift in consumer awareness resulting in an increased preference for organic plant produce [17]. Thus, the high production of okra the world over should be utilized to some extent in the large-scale production of okra-based nutraceuticals, which could also be used to alleviate the problem of malnutrition in underdeveloped countries.

## 8. Safety and Efficacy

Okra is a food crop, and its elongated, edible pods are mostly harvested during the immature stages and eaten primarily as a vegetable dish. Other parts of okra—namely, the flowers and buds—are also palatable [112]. The premature pods are commonly ingested in vegetable dishes and are also consumed dried, marinated in salads, fried, raw, or boiled, along with various other ingredients [10]. An average fresh okra pod is estimated to contain approximately 740 IU of vitamin A. Okra seeds also provide a rich source of edible oil, constituting up to 22% of the biomass [10]. Toxicological reports have suggested that the fruit and seeds of okra are nontoxic at normal levels of consumption. Few reports have substantiated the safety and efficacy of okra extracts in controlled human trials. *Abelmoschus manihot*, a single medicament of traditional Chinese medicine widely used to treat kidney disease, was evaluated in the form of a huangkui capsule, 2.5 g, three times per day, losartan potassium, 50mg/d, or a combined treatment—a huangkui capsule at 2.5 g, three times per day, was combined with losartan potassium, 50mg/d. The duration of the intervention was 24 weeks. The results were evaluated in the form of changes within the mean baseline urine protein excretion and changes in the estimated glomerular filtration rate (eGFR) from the baseline after treatment. The study proved the efficacy of *Abelmoschus manihot* as a promising therapeutic for patients with primary kidney disease (chronic kidney disease stages 1 and 2) with moderate proteinuria [34].

Similarly, IQP-AE-103 is a combination of dehydrated powder of okra (*A. esculentus* (L.) Moench) pods and inulin; a heterogeneous mixture of fructose polymers extracted from chicory roots was used to evaluate the efficacy and tolerability/safety of IQP-AE-103 on body weight reduction in overweight-to-moderately obese adults. A beneficial effect of IQP-AE-103 on the lipid metabolism was also demonstrated in the subgroup of subjects with baseline total cholesterol levels above 6.2 mmol/L. The study indicated that IQP-AE-103 could be an effective and safe weight loss intervention; the trial was registered with NCT03058367 [140]. However, currently, a trial to evaluate the glycemic evaluation of okra seed noodles is currently active, although not recruiting; see clinicaltrials.gov (ClinicalTrials.gov Identifier: NCT03990844). Data on the safety and toxicity of okra fruits are limited. Therefore, further clinical studies and research is required on this edible medicinal plant in the context of nutraceutical and functional food development, food excipients, drug discovery, and development [9,117].

## 9. Future Perspectives and Conclusions

The attributes of the different edible okra parts discussed in this review highlight the nutritional relevance of okra and its nutraceutical potential for providing health benefits to humans. Okra is a cost-effective and economically affordable natural source with ample reservoirs of carbohydrates, proteins, fatty acids, vitamins, fiber, and minerals, with various other bioactive phytochemicals that are important for human well-being. Although the potential benefits of okra on different chronic disorders have been scientifically explored to some extent, several aspects, such as the pharmacokinetics and bioavailability of okra, as well as its specific mechanisms of action on different diseases, need further investigation. Such a knowledge gap may have arisen due to the complex etiology of diseases, along with different related factors aiding the diseases. Owing to the nutritional composition of okra, it can be potentially used to compensate for the problem of malnutrition in underdeveloped countries across the globe. Moreover, the formulation of okra-based nutraceuticals could prove to be beneficial, since it is easily available and inexpensive. Therefore, further studies should focus on the development of functional foods, nutraceuticals, or drugs from okra components.

## Figures and Tables

**Table 1:** Nutritional composition of raw okra per 100 g of serving. Data reported from USDA (United States Department of Agriculture) SR-21.

S. No.	Dietary Constituents	Amount Per Serving	%DV *
1.	Total calories	130 kJ	2
2.	Total carbohydrates	7 g	2
3.	Total protein	2.0 g	4
4.	Dietary fiber	3.2 g	13
5.	Starch	0.3 g	-
6.	Sugar	1.2 g	-
7.	Total fat	0.1 g	-
8.	Trans-fat	-	-
9.	Saturated fat	0.0 g	0
10.	Cholesterol	0.0 mg	0
11.	Total omega-3 fatty acids	0.001 g	-
12.	Total omega-6 fatty acids	0.026 g	-
13.	Phytosterols	0.024 g	-

* Indicates the daily limit percentage for adults and children aged ≤ four years [20].

**Table 2:** Mineral intake per serving of 100 g of okra. Data reported from USDA SR-21.

S. No.	Minerals	Amount Per Serving	%DV *
1.	Potassium	303 mg	9
2.	Calcium	81.0 mg	8
3.	Phosphorus	63.0 mg	6
4.	Magnesium	57.0 mg	14
5.	Copper	0.1 mg	5
6.	Selenium	0.7 µg	1
7.	Manganese	1.0 mg	50
8	Zinc	0.6 mg	4
9.	Sodium	8.0 mg	0
10.	Iron	0.8 mg	4

* Indicates the daily limit percentage for adults and children aged ≤ 4 years [20].

**Table 3:** Different bioactive components derived from okra showing their therapeutic benefits on human health, along with their mechanisms of action.

Bioactive Components	Therapeutic Benefits	Mechanisms of Action	Reference
Polysaccharide	Antidiabetic	It helps to lower body weight and glucose levels, improve glucose tolerance, and decrease the total serum cholesterol levels in high-fat diet-fed C57BL/6 mice.	[79]
Rhamnogalacturonan	Antidiabetic	Hypoglycemic effect,	[80]
Lectins	Anticancer	Arrest the cell cycle and activate the caspase cascades.	[81]
		Inhibit cellular proliferation in human breast cancer in vitro.	[70]
Pectin	Anticancer	Involved in cell adhesion, growth, and survival, as well as tumor development and cancer prevention therapy.	[82,83,84]
	Antiproliferative and proapoptotic	Induce apoptosis and inhibit cellular proliferation.	[85]
Pectin	Lower bad cholesterol	Okra promotes cholesterol degradation and inhibits the production of fat in the body. It lowers bad cholesterol by altering the bile production in the intestines. This helps in eliminating the clots and deposited cholesterol.	[86]
Polyphenolic compounds	Antioxidant	Extract exhibits a strong DPPH radical scavenging activity and reducing power.	[87]
Quercetin 3-O-glucosyl (1→6) glucoside (QDG) and quercetin 3-O-glucoside (QG)	Antioxidant	Excellent reducing power and free radical scavenging capabilities, including DPPH, superoxide anions, and hydroxyl radicals.	[88]
Vitamin C, calcium, iron, manganese, and magnesium	Antioxidant	Eliminating free radicals.	[86]
Quercetin derivatives and epigallocatechin	Antioxidant	Inhibitory effects on the generation of reactive oxygen species (ROS).	[54]
Polysaccharide	Metabolic disorders	Inhibition of LXR and PPAR signaling.	[79]
Polyphenolic compounds,	Antioxidant	Perform the function of capturing free radicals and stopping the chain reactions.	[89]
Vitamin A; B vitamins (B1, B2, B6); and vitamin C and traces of zinc, calcium, folic acid, and fiber	Pregnancy benefits	Folates prevent miscarriages. They are also beneficial in the formation of the neural tube of the fetus and protect these tubes, preventing defects. This helps prevent birth defects like spina bifida and can even stop constipation during pregnancy.	[86]
Polyphenols like catechin and flavonoids like quercetin possess	Antifatigue effects	Decreased the levels of blood lactic acid (BLA) and BUN in the blood; MDA in the liver; and increased the levels of HG, SOD, and GSH in the liver during fatigue recovery, which proved that OSD could alleviate physical fatigue and promote recovery.	[51]
Probiotics	Gut bacteria-friendly	Biosynthesis of the vitamin B complex.	[86]
Glutathione	Detoxify liver, antioxidant	The slimy substance in okra contains substances that bind bile acid and cholesterol to detoxify the liver.	[86]
Mucilaginous	Ulcer treatment	The slimy stuff in okra is alkaline. This helps in neutralizing the acid. Additionally, it provides a protective coating within the digestive tract, which speeds up the healing process of peptic ulcers.	[86]
Mucilaginous with fiber	Relieves and prevents constipation	Bind toxins and lubricates the large intestines. This ensures effortless and normal bowel movement due to its natural laxative property.	[86]	
Vitamin K and C	Bone health and essential for the blood-clotting process. It also helps restore bone density and prevent osteoporosis.	Several mechanisms are suggested by which vitamin K can modulate bone metabolism. Besides the gamma-carboxylation of osteocalcin, a protein believed to be involved in bone mineralization, there is increasing evidence that vitamin K also positively affects the calcium balance, a key mineral in bone metabolism.	[86,90]
Vitamin A, along with antioxidant contents such as lutein, xanthein, and carotenes	Improves vision	Okra contains beta-carotenes (precursor of vitamin A), xanthin, and lutein, all with antioxidant properties preventing eye problems like cataract and glaucoma.	[86]
Glycosylated compounds	Antibacterial activity	Inhibit the adhesion of *Helicobacter pylori* to the human gastric mucosa.	[78]
Rhamnogalacturonan Polysaccharides	Antiadhesive properties	Interrupt the adhesion of *H. pylori* to human stomach tissues via interfering with the outer membrane proteins.	[78,91,92,93]
Polyphenols and flavonoids (okra seeds)	Antifatigue	Reduce the levels of BLA and BUN, enhancing hepatic glycogen storage and the promoting antioxidant ability by lowering the MDA level and increasing the SOD and GSH-PX levels.	[51]

**Table 4:** Health benefits of various phytochemicals present in okra and its various parts.

Bioactive Components	Part	Health Benefits	Reference
Polyphenols Carotene	Pod	Important for eyesight, along with healthy skin.	[78,108]
Folic acid		Beneficial for fetus development.	[1,109]
Thiamine		Improves the nervous system, brain, heart, stomach, muscles, and intestine functions.	[8,110]
Riboflavin		Needed for growth and overall good health.	[111,112]
Niacin		Keeps our nervous system, digestive system, and skin healthy.	[108,113]
Vitamin C		Helps in the overall growth of the body and tissue repair.	[1,114]
Oligomeric catechin	Seed	Prevents and is used for treating chronic ailments, e.g., cardiovascular diseases and cancer.	[10,115]
Flavonol derivatives		Improves vascular health, leading to a reduced risk of diseases.	[116,117]
Lysine		Improves calcium absorption and retention.	[118,119]
Palmitic acid		An important constituent of the cell membrane, with a critical role in protein palmitoylation and palmitoylated signal molecules.	[120,121]
Oleic acid		Decreases the cholesterol levels and prevents heart diseases.	[122,123]
Linoleic acid		Improves cardiovascular health.	[88,124]
Carbohydrate	Roots	Prime energy source and fuel for the brain, kidney, heart, and muscles.	[125,126]
Flavonoids		Exhibits substantial anticancer, antioxidant, anti-inflammatory, and hepatoprotective activities.	[10,127]
Minerals	Leaves	Helps in overall growth and body development.	[128,129]
Tannins		Accelerate blood clotting, reduces the serum lipid and blood pressure, and modulates the immune responses.	[130,131,132]

## Data Availability

All data generated or analyzed during this study are included in this article.
